# Supplementary material for: Pivotal Role of AKT2 during Dynamic Phenotypic Change of Breast Cancer Stem Cells
Source: Cancers (Basel). 2019 Jul 26;11(8):1058. doi: 10.3390/cancers11081058 (PMC6721305; doi:10.3390/cancers11081058)
Supplement: Supplementary file 1 [file cancers-11-01058-s001.pdf]

Figure S1

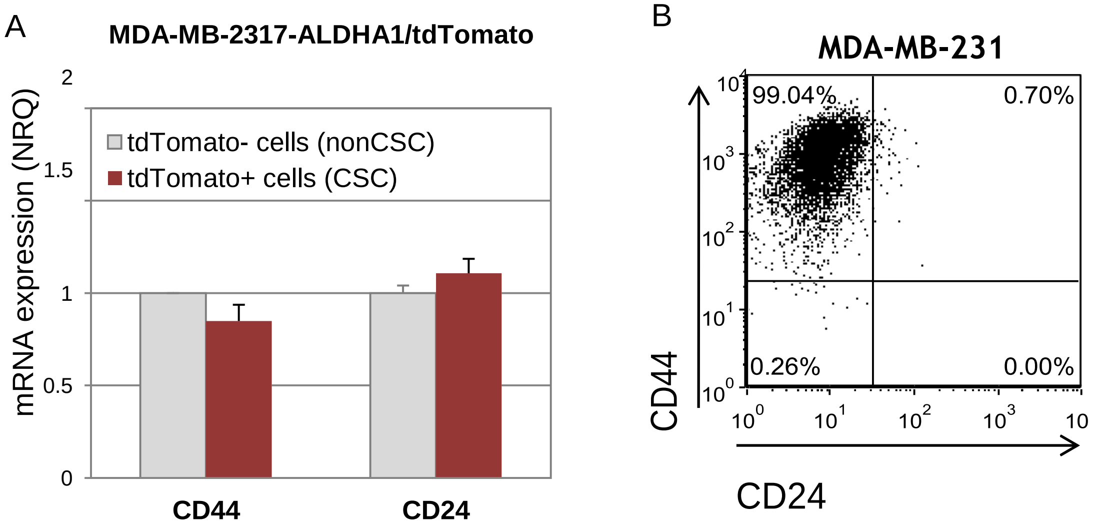


**Figure S1. CD44 and CD24 expression in MDA-MB-2317-ALDHA1/tdTomato cells**.
qPCR quantification of *CD44* and *CD24* did not revealed any differences between MDA-MB-231 CSC-like cells (tdTomato$^{+}$) versus MDA-MB-231 nonCSC (tdTomato$^{-}$). Results are expressed as NRQ (relative normalized quantities) mean ± SEM ($n \geq 3$) (**A**).Expression of CD44 was high, and CD24 was low in MDA-MB-231 cells according to its mesenchymal-stem like (MSL) characteristics (**B**).

Figure S2

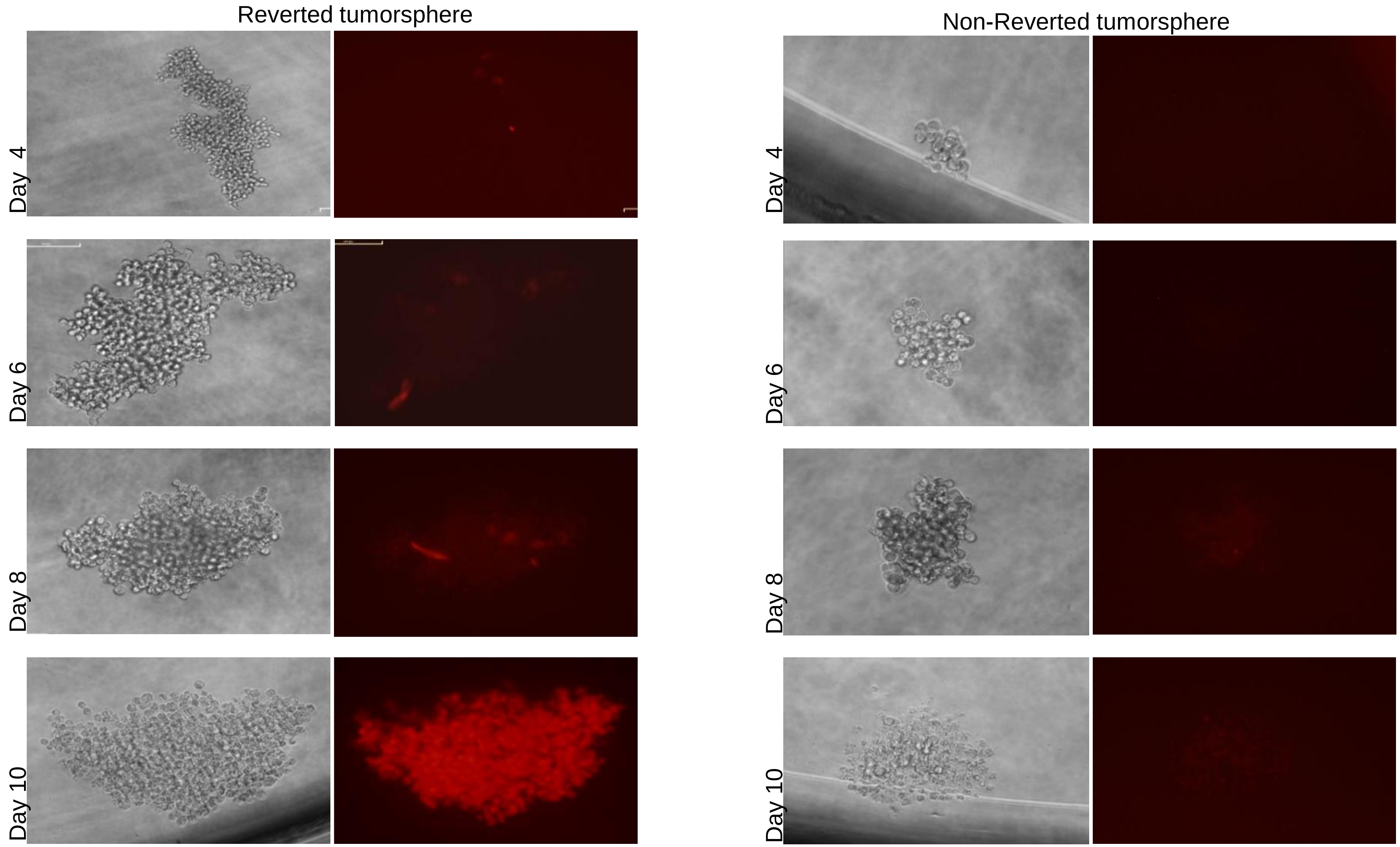


**Figure S2. MDA-MB-231 mammosphere culture**.

MDA-MB-231 tdTomato$^{-}$ (non-CSC) were placed in low attachment plate in FBS-free culture medium, and were cultured during 10 days. The mammosphere formation was followed by fluorescent microscopy. A sudden general switch to tdTomato$^{+}$ phenotype of tumorsphere was observed. Tumorsphere that did not switch phenotype did not survive in low attachment long-term culture.

Figure S3

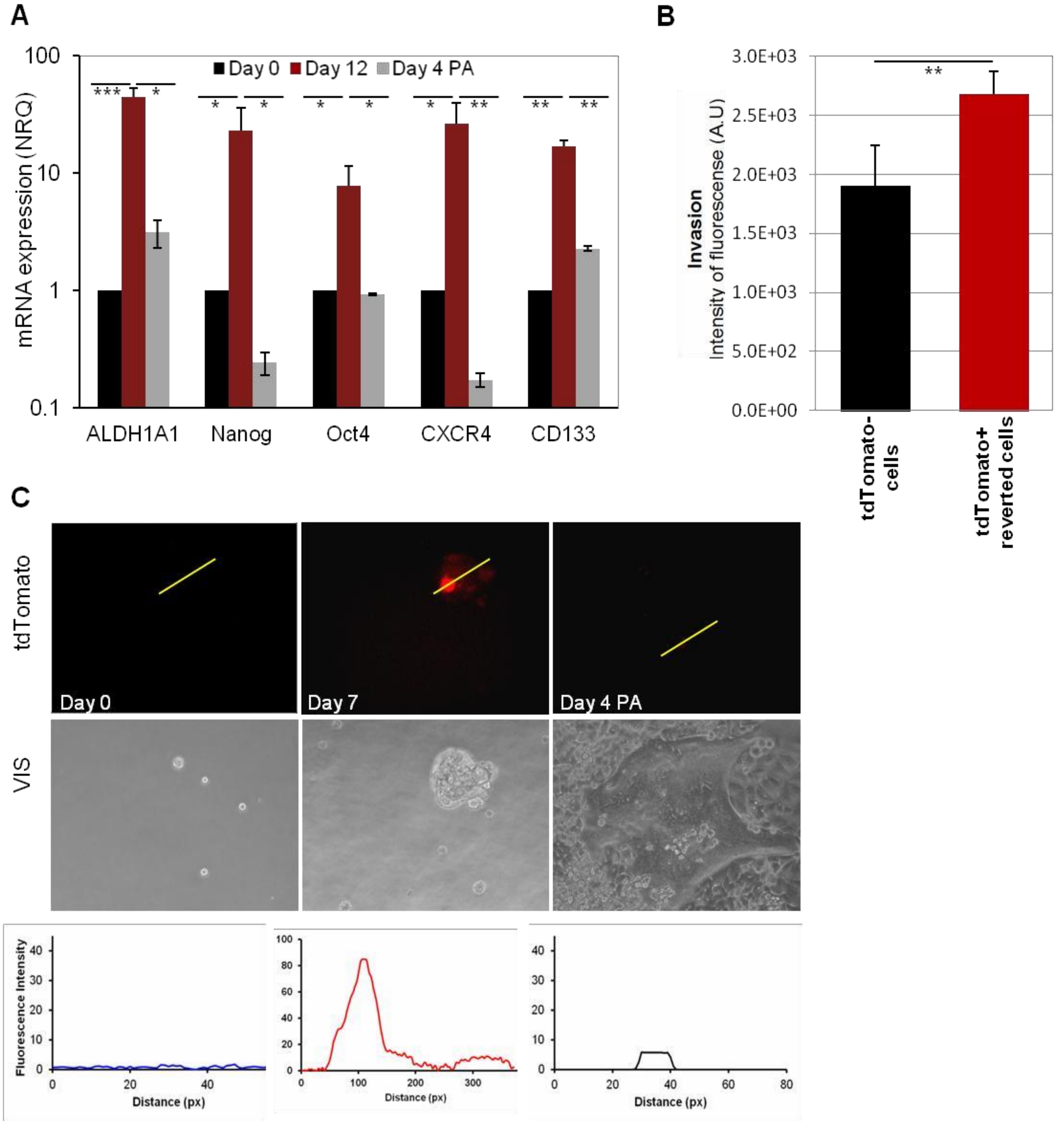


**Figure S3. HCT8 reversion process**

The reversion process was confirmed by change in stemness genes expression profile by qPCR. Results are expressed as NRQ (relative normalized quantities) mean ± SEM ($n \geq 3$); (* $p < 0.05$; ** $p < 0.01$, *** $p < 0.001$); **(A)** and by increase of invasion ability of reverted HCT-8 CSCs compared to initial tdTomato$^-$ (non-CSCs) **(B)**. In addition, the reversion was monitored over the time by fluorescent microscopy **(C)**.

Figure S4

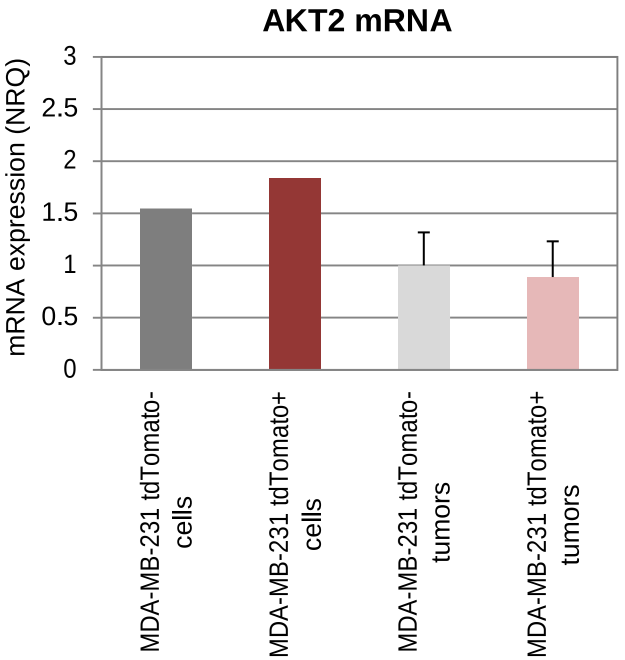


**Figure S4. *AKT2* mRNA expression in the tumor derived from MDA-MB-231 CSC-like and nonCSC cells.**
*AKT2* mRNA was not altered in CSC-like CSCs compared to nonCSC like MDA-MB-231 cells. Similarly, *AKT2* mRNA expression was not altered in the tumor derived from CSC-like cells nonCSC MDA-MB-231 cells, respectively. Results are expressed as NRQ (relative normalized quantities) mean ± SEM ($n \geq 3$).

Figure S5

A

| log2ratio_ MCF7 CSC vz. nonCSC | log2ratio_ MDA231 CSC vz. nonCSC | log2ratio_CSC vz. nonCSC | log2ratio_MDA231 vz. MCF7 | GeneNamec | FC_MCF7 CSC vz. nonCSC | FC_MDA231 CSC vz. nonCSC | FC_CSC vz. nonCSC | P.Value_CSC vz. nonCSC | P.Value_CSC vz. nonCSC | FC_MDA231 vz. MCF7 | P.Value_MDA231 vz. MCF7 | P.Value_MDA231 vz. MCF7 | Systematic Name |
|---|---|---|---|---|---|---|---|---|---|---|---|---|---|
| | | | | AKT2 | 1.11 | -1.06 | 1.02 | 9.09E-01 | 1.00E+00 | 2.48 | 7.71E-04 | 5.74E-03 | NM_001626 |
| | | | | AKT1 | 1.01 | -1.02 | -1.01 | 9.76E-01 | 1.00E+00 | -2.50 | 1.73E-05 | 3.19E-04 | NM_005163 |
| | | | | AKT3 | -1.29 | 1.11 | -1.07 | 8.86E-01 | 1.00E+00 | 17.51 | 9.08E-07 | 3.72E-05 | NM_181690 |

B

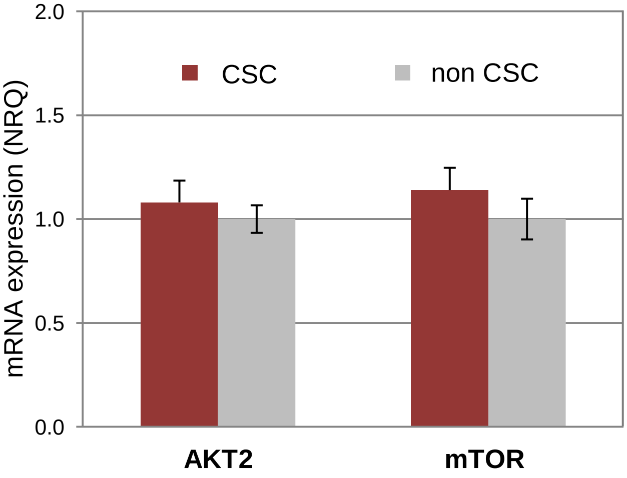


**Figure S5.** ***AKT1*, *AKT2*, *AKT3* mRNA expression in MDA-MB-231 CSC.**

*AKT1*, *AKT2*, *AKT3* mRNA was not altered in CSC-like CSCs compared to nonCSC like MDA-MB-231 cells according microarray examination. Results are expressed as NRQ (relative normalized quantities) mean ± SEM ($n \geq 3$) (**A**). These results, regarding *AKT2* and *mTOR* expression, were confirmed further by qPCR (**B**).

Figure S6

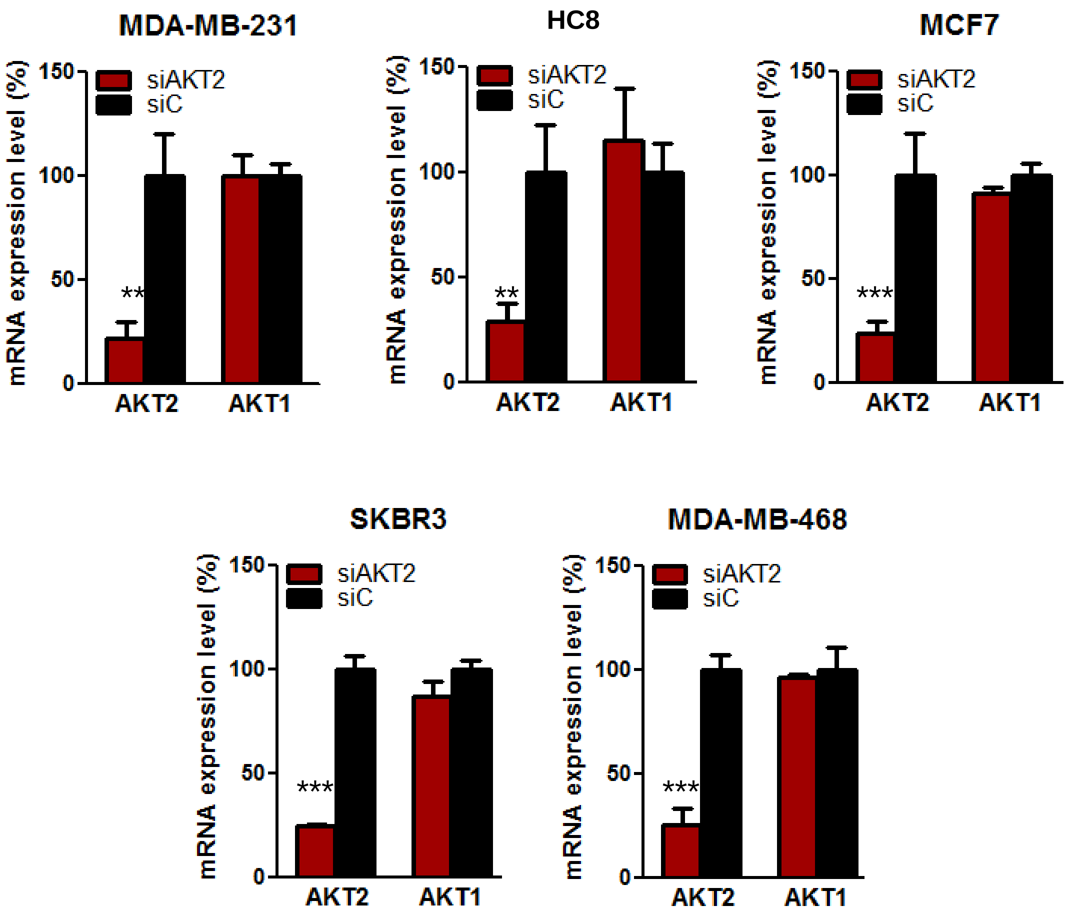


**Figure S6.** ***AKT2*** **downregulation and gene expression profile of various mesenchymal and epithelial breast and colon cancer cells**.

qPCR quantification of *AKT2* and *AKT1* expression levels shows, that knockdown of *AKT2* using specific siRNA against *AKT2* (siAKT2) was specific. No effect was observed on *AKT1* gene or when scrambled siRNA (siC) was employed. Results are expressed as NRQ (relative normalized quantities) mean ± SEM ($n \geq 3$); (* $p < 0.05$; ** $p < 0.01$, *** $p < 0.001$).

Figure S7

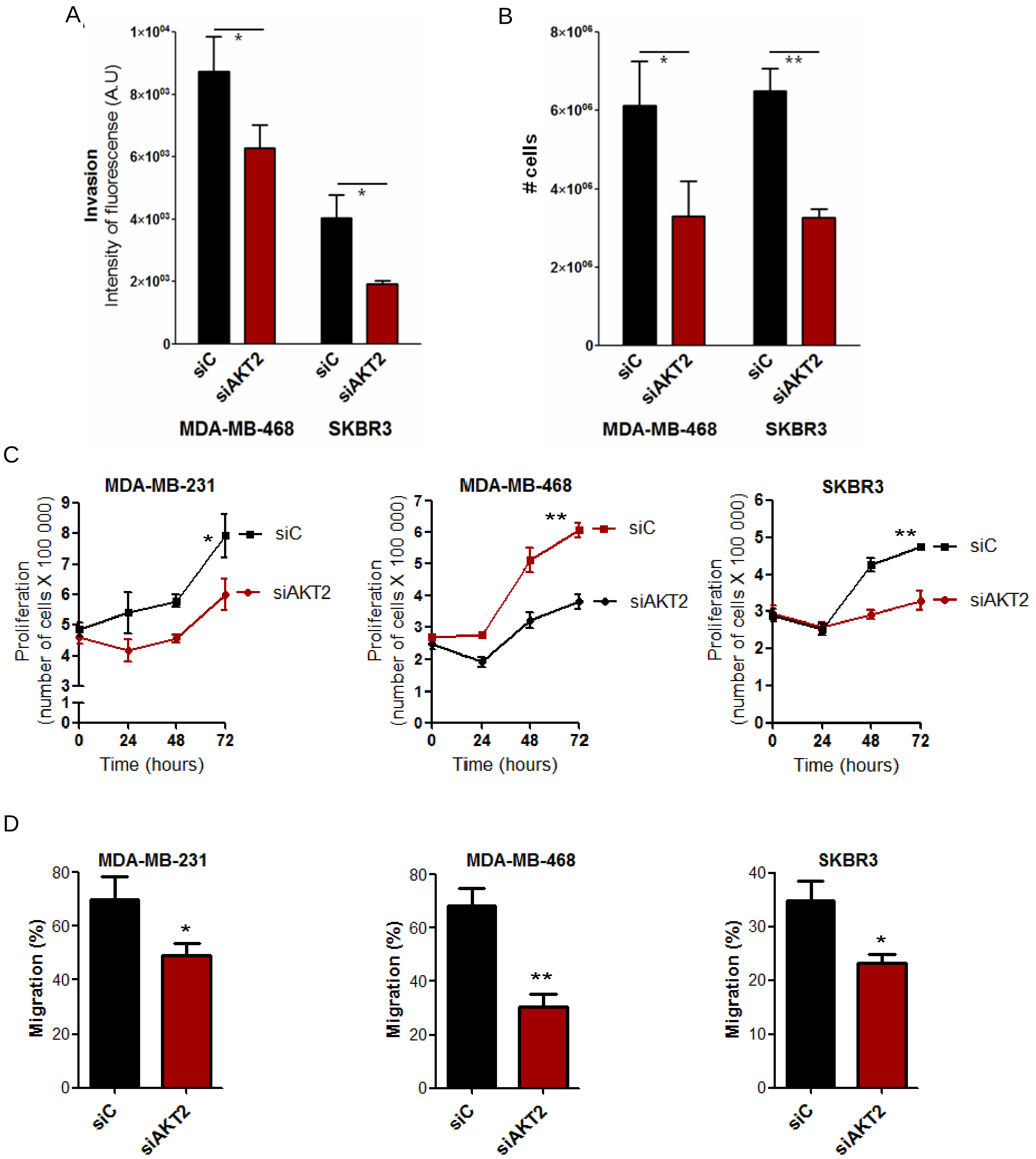


**Figure S7.** Effect of *AKT2* silencing on Invasion **(A)** Transformation **(B)** Proliferation **(C)** and migration **(D)**. All results are expressed as mean ± SEM ($n \geq 3$) compared to siC transfected cells; (* $p < 0.05$; ** $p < 0.01$, *** $p < 0.001$).

Figure S8

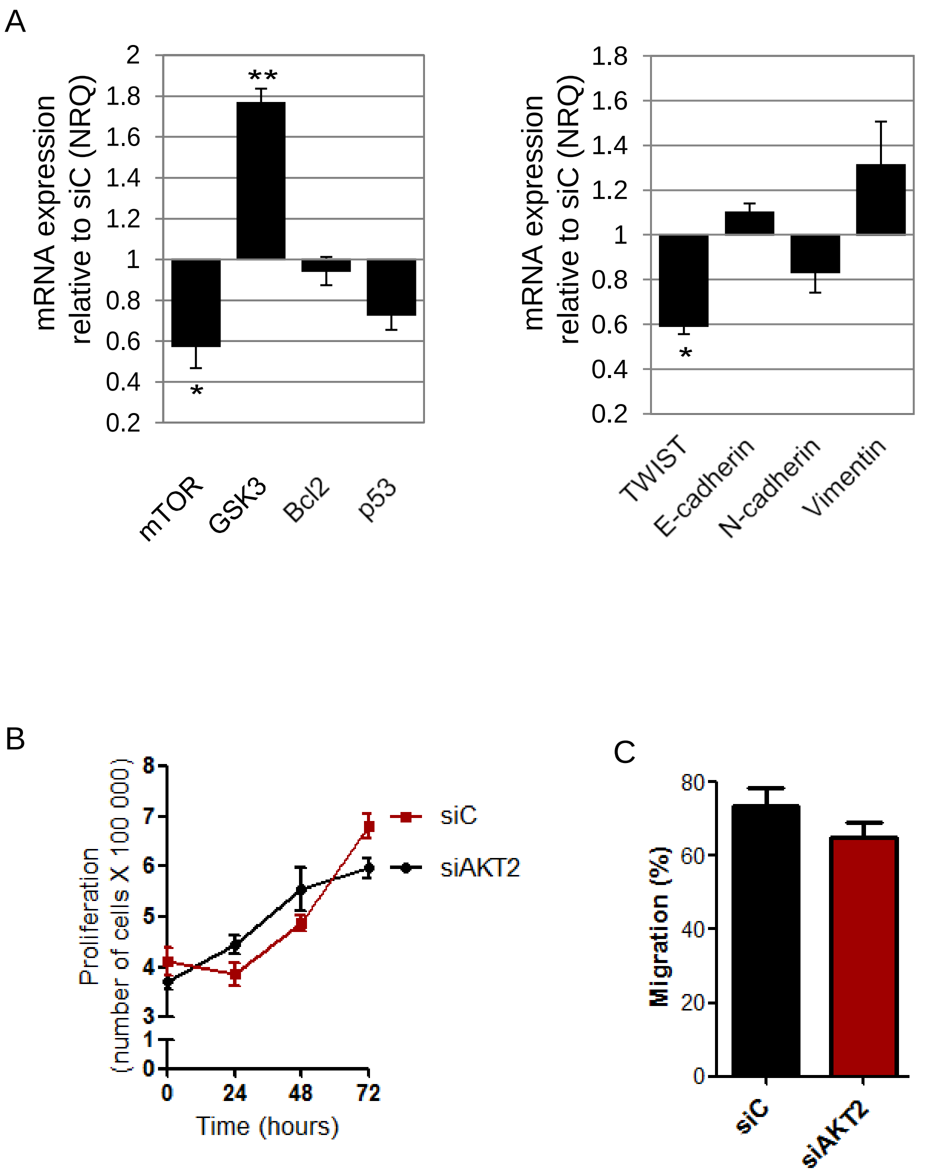


**Figure S8. Effect of *AKT2* silencing in parental MCF7 epithelial cells.**
We observed significant change of *mTOR*, *GSK3* and *TWIST* expression by qPCR after the treatment with siAKT2 compared to a treatment with siC in MCF7 cells. No others studied genes were altered. Results are expressed as NRQ (relative normalized quantities) mean ± SEM ($n \geq 3$); (* $p < 0.05$; ** $p < 0.01$, *** $p < 0.001$) **(A).** There was no significant change in cell proliferation capacity after *AKT2* silencing, compared to a treatment with siC in MCF7 cells **(B)**. Number of migratory cells after treatment with siAKT2 was no altered compared to a treatment with siC in MCF7 cells **(C)**.

Figure S9

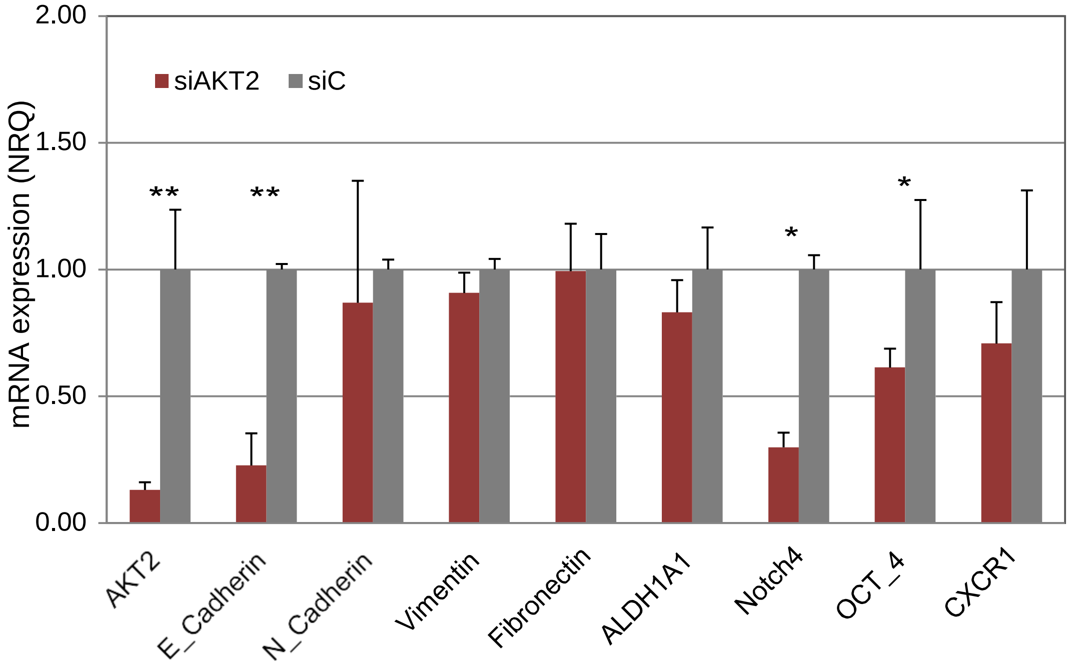


**Figure S9. Gene expression after AKT2 silencing in MDA-MB-231 cell line**.

We observed also down-regulation of *E-CADHERIN* and some stem cell genes (*OCT4*, *NANOG*) after *AKT2* silencing in MDA-MB-231 cells, even though the expression of *N-CADHERIN*, *VIMENTIN* and *FIBRONECTIN* was not changed in these mesenchymal cell line, conforming tide connection between EMT and CSC phenotype. Results are expressed as NRQ (relative normalized quantities) mean ± SEM ($n \geq 3$); (* $p < 0.05$; ** $p < 0.01$, *** $p < 0.001$).

**Supplemental Experimental Procedures**

**Supplementary Text 1. Cell culture**

Breast (MCF-7, MDA-MB-231, MDA-MB-468 and SKBR3) and colon (HCT8 and HCT116) cancer cell lines were obtained from American Type Culture Collection (ATTC, LGC Standards, Barcelona, Spain) (Table S1). MDA-MB-468, MDA-MB-231, HCT-8 and HCT 116 cells were cultured in RPMI medium (Lonza, Barcelona, Spain) supplemented with 10 % fetal bovine serum (FBS) (Lonza, Barcelona, Spain), 1% penicillin-streptomycin, 1% 2 mM L-Glutamine, 1% non- essential amino acids and 1% of sodium pyruvate (Life Technologies, Madrid, Spain); SKBR3 cells in McCoy's medium (Life Technologies, Madrid, Spain) supplemented with a 10% FBS and 1% penicillin-streptomycin; MCF-7 cells were cultured in DMEM F-12 medium (Life Technologies, Madrid, Spain) supplemented with 10% FBS and 1% penicillin-streptomycin. Blasticidin (10 μg/mL) (Life Technologies, Madrid, Spain) was used as a selective antibiotic for ALDH1/tdTomato cell lines. All cell lines were maintained in an atmosphere with 5% of $CO_2$ at 37$^0$C.

**Supplementary Text 2. Microarray performance**

Cells were sorted according to tdTomato and DAPI expression in a FACS Aria cell sorter (BD Biosciences, Spain). In total 8 RNA samples, comparing 2 technical replicates of tdTomato-positive and tdTomato-negative samples (corresponding to CSC and non-CSC) isolated from two different cell lines (MCF7-ALDH1A1:tdTomato, MDA-MB-231-ALDH1A1:tdTomato) were analyzed by microarray. For this, 100 ng of total RNA was labeled using LowInputQuick Amp Labeling kit v6.5 (Agilent, Santa Clara, CA, USA) following manufacturer instructions. The labeled cRNA was hybridized to the Agilent SurePrint G3 Human gene expression 8 × 60K microarray (ID039494) according to the manufacturer's protocol. The arrays were scanned on an Agilent G2565CA microarray scanner at 100% PMT and 3 mm resolution. Raw data was taken from the Feature Extraction output files and was corrected for background noise using the normexp method. To assure comparability across samples we used quantile normalization. Differential expression analysis was carried out on non control probes with an empirical Bayes approach on linear models (limma), applying a paired test. Results were corrected for multiple testing according to the False Discovery Rate (FDR) method.

**Table S1.** List of the cell lines used in this study and their phenotype.

| Name | ATCC® number | Origin | Phenotype | AKT2 expression | TWIST expression | mTOR expression |
|---|---|---|---|---|---|---|
| MDA-MB-231 | HTB-26™ | Breast (Triple Negative) | Mesenchymal | Yes | No | Yes |
| MDA-MB-468 | HTB-132™ | Breast (Triple Negative) | Mesenchymal | Yes | Yes | Yes |
| MCF7 | HTB-22™ | Breast (ER+) | Epithelial | Yes | Yes | Yes |
| SKBR3 | HTB-30™ | Breast (HER2+) | Mesenchymal | Yes | Yes | Yes |
| HCT8 | CCL-244™ | Colon | Mixt | Yes | No | Yes |

ATCC—american type culture collection (https://www.lgcstandards-atcc.org); ER—estrogen receptor; HER2—human epidermal growth factor receptor 2; AKT2—Serine/Threonine Kinase 2; TWIST—twist family bHLH transcription factor 1; mTOR—mechanistic target of rapamycin kinase.

**Table S2.** List of the primers used in the study and their sequences.

| Oligo name | Oligo Sequence (5′ –3′) |
|---|---|
| *βACTIN* F | CATCCACGAAACTACCTTCAACTCC |
| *βACTIN* R | GAGCCGCCGATCCACAC |
| *AKT1* F | GGCTCCCCTCAACAACTTCTC |
| *AKT1* R | ACATGGAAGGTGCGTTCGA |
| *AKT2* F | CAAGGATGAAGTCGCTCACACA |
| *AKT2* R | GAACGGGTGCCTGGTGTTC |
| *E-CADHERIN* F | TACGCCTGGGACTCCACCTA |
| *E-CADHERIN* R | CCAGAAACGGAGGCCTGAT |
| *FIBRONECTIN* F | AAGGTTCGGGAAGAGGTTGTTAC |
| *FIBRONECTIN* R | CGGCATAATGGGAAACTGTGT |
| *GADPH* F | ACCCACTCCTCCACCTTTGAC |
| *GADPH* R | CATACCAGGAAATGAGCTTGACAA |
| *GSK3b* F | CTGCTTCAACCCCCACAAAT |
| *GSK3b* R | GATGCAGAAGCAGCATTATTGG |
| *mTOR* F | CCTTAACGTCATTCGAGTCTGTGA |
| *mTOR* R | TCACAAAGGACACCAACATTCC |
| *N-CADHERIN* F | TCAAAGCCTGGAACATATGTGATG |
| *N-CADHERIN* R | GTATACTGTTGCACTTTTTCTCGTACAA |
| *NOTCH4* F | CCAGCCAGGCACGTCAG |
| *NOTCH4* R | TGTAGCGATTAATGCCATCCAT |
| *OCT4* F | CCTGCACCGTCACCCCT |
| *OCT4* R | GGCTGAATACCTTCCCAAATAGAAC |
| *P53* F | GAAGAAACCACTGGATGGAGAATATT |
| *P53* R | CAGCTCTCGGAACATCTCGAA |
| *TWIST* F | GCAGGGCCGGAGACCTAG |
| *TWIST* R | TTTTTAGTTATCCAGCTCCAGAGTCTCTA |
| *VIMENTIN* F | CTCCGGGAGAAATTGCAGG |
| *VIMENTIN* R | AGACGTGCCAGAGACGCATT |

**Table S3.** List of primary antibodies used in the study and their specifications.

| Primary antibody | Source | Host | Dilution | Incubation |
|---|---|---|---|---|
| AKT2 | Cell Signalling | Rabbit | 1:1000 | ON, 4 $^0$C |
| TWIST | Abcam | Mouse | 1:500 | ON, 4 $^0$C |
| βACTIN | Abcam | Rabbit | 1:1000 | 1 h, RT |
| mTOR | Cell Signalling | Mouse | 1:1000 | ON, 4 $^0$C |

ON—overnight; RT—room-temperature; AKT2—Serine/Threonine Kinase 2; TWIST—twist family bHLH transcription factor 1; mTOR—mechanistic target of rapamycin kinase.
